# Dimethyl fumarate as a promising therapeutic candidate for virus-associated myelopathy

**DOI:** 10.1093/brain/awaf447

**Published:** 2026-02-05

**Authors:** Takashi Yoshida, Satoshi Nozuma, Masakazu Tanaka, Mika Dozono, Daisuke Kodama, Toshio Matsuzaki, Tomoko Kondo, Ryuji Kubota, Hiroshi Takashima

**Affiliations:** Department of Neurology and Geriatrics, Kagoshima University Graduate School of Medical and Dental Sciences, Kagoshima 890-8520, Japan; Department of Neurology and Geriatrics, Kagoshima University Graduate School of Medical and Dental Sciences, Kagoshima 890-8520, Japan; Division of Neuroimmunology, Joint Research Center for Human Retrovirus Infection, Kagoshima University, Kagoshima 890-8544, Japan; Department of Neurology and Geriatrics, Kagoshima University Graduate School of Medical and Dental Sciences, Kagoshima 890-8520, Japan; Division of Neuroimmunology, Joint Research Center for Human Retrovirus Infection, Kagoshima University, Kagoshima 890-8544, Japan; Division of Neuroimmunology, Joint Research Center for Human Retrovirus Infection, Kagoshima University, Kagoshima 890-8544, Japan; Division of Translational Medicine, Joint Research Center for Human Retrovirus Infection, Kagoshima University, Kagoshima 890-8544, Japan; Division of Neuroimmunology, Joint Research Center for Human Retrovirus Infection, Kagoshima University, Kagoshima 890-8544, Japan; Department of Neurology and Geriatrics, Kagoshima University Graduate School of Medical and Dental Sciences, Kagoshima 890-8520, Japan

**Keywords:** neuroinflammation, T-cell proliferation, cytokine suppression, drug repositioning

## Abstract

Human T-cell lymphotropic virus type 1 (HTLV-1)-associated myelopathy/tropical spastic paraparesis (HAM/TSP) is a chronic, progressive neuroinflammatory disease with no effective treatment. In this study, we investigated whether dimethyl fumarate (DMF), an immunomodulatory agent approved for treating multiple sclerosis, exerts therapeutic effects relevant to HAM/TSP.

Peripheral blood mononuclear cells (PBMCs) from 16 people living with HAM/TSP were used to evaluate the effects of DMF on cell viability, spontaneous proliferation, inflammatory cytokine production and HTLV-1 proviral load (PVL).

DMF significantly inhibited lymphocyte proliferation in a concentration-dependent manner, with reductions of 42.1% at 10 µM, 56.3% at 25 µM, 60.6% at 50 µM and 69.9% at 100 µM. This suppressive effect was particularly evident in CD8+ T cells, CD4+ T cells and HTLV-1-infected CD4+ T cells. Furthermore, DMF reduced the production of interleukin (IL)-6, tumor necrosis factor-alpha (TNF-α) and interferon-gamma (IFN-γ) released from these proliferating cells. A reduction in PVL was also observed in a subset of *ex vivo* PBMC cultures derived from individuals with HAM/TSP exhibiting high viral proliferative activity.

These results suggest that DMF suppresses pathogenic immune activation in HAM/TSP and may therefore represent a promising therapeutic candidate for this disabling neuroinflammatory disorder.

## Teaser

Human T-cell lymphotropic virus type 1-associated myelopathy/tropical spastic paraparesis is a debilitating neuroinflammatory disease. Yoshida *et al.* show that dimethyl fumarate inhibits the proliferation of infected cells and inflammatory cytokine production in patient samples, suggesting potential as an immunomodulatory therapy.

## Introduction

Human T-cell lymphotropic virus type 1 (HTLV-1) is a retrovirus that establishes lifelong infection in humans, with an estimated global prevalence of 5 to 10 million individuals.^1^ The majority of infected individuals remain asymptomatic carriers (AC); however, approximately 2%–5% develop adult T-cell leukaemia/lymphoma (ATL),^2^ and 0.25%–3.8% develop HTLV-1-associated myelopathy/tropical spastic paraparesis (HAM/TSP).^3,4^ HAM/TSP is a chronic, progressive neuroinflammatory disorder characterized by spastic paraparesis, urinary dysfunction and sensory impairment in the lower extremities. Disease progression can lead to severe disability, including wheelchair dependence or a bedridden state, and HAM/TSP is therefore considered an intractable neurological condition.^5^

The pathogenesis of HAM/TSP is characterized by chronic inflammation of the spinal cord, primarily driven by an excessive immune response targeting HTLV-1-infected T cells.^6,7^ HTLV-1 preferentially infects CD4+ T cells, which undergo proliferation and migrate into the CNS.^8^ During this process, they induce the activation and expansion of HTLV-1-specific cytotoxic CD8+ T lymphocytes (CTLs), which in turn promote the production of inflammatory cytokines and chemokines.^9,10^ This immune cascade contributes to bystander damage of surrounding neurons and glial cells, ultimately leading to progressive neurological impairment.^11^

Although various therapeutic approaches have been proposed, no established treatment currently exists for HAM/TSP.^12^ Glucocorticoids are commonly used to suppress the excessive immune responses characteristic of the disease, and have been shown to improve motor symptoms and slow disease progression.^13,14^ However, various adverse effects limit long-term use, often leading to treatment discontinuation. Interferon (IFN)-α, which possesses both immunomodulatory and antiviral properties, has demonstrated clinical efficacy in randomized double-blind controlled trials;^15^ nevertheless, its use is limited by a high incidence of adverse events and a lack of established long-term benefits. Meanwhile, antiviral therapies targeting HTLV-1 reverse transcriptase have failed to show efficacy in clinical trials.^16,17^ In addition, valproic acid (VPA), a histone deacetylase inhibitor, has been investigated as a therapeutic candidate for HAM/TSP. A clinical trial demonstrated that VPA transiently activated HTLV-1 expression, leading to a reduction in HTLV-1 proviral load (PVL) *in vivo*, though it did not significantly alter long-term disability.^18,19^ More recently, the humanized anti-C-C chemokine receptor type 4 (CCR4) monoclonal antibody mogamulizumab was shown to reduce HTLV-1 PVL as well as levels of neopterin and C-X-C motif chemokine ligand (CXCL)10 in the cerebrospinal fluid of people living with HAM/TSP;^20^ however, these effects have not resulted in significant clinical improvement.^21^ Given the limitations of current therapeutic options, there is an urgent need to develop novel treatment strategies for HAM/TSP.

Dimethyl fumarate (DMF), an oral immunomodulatory agent approved for the treatment of multiple sclerosis (MS), exerts both anti-inflammatory and neuroprotective effects through multiple mechanisms.^22-25^ DMF suppresses T-cell activation and proliferation, particularly attenuating the expansion of autoreactive CD4+ and CD8+ T cells involved in CNS inflammation. It also activates the nuclear factor erythroid 2-related factor 2 (Nrf2) signalling pathway, which induces cytoprotective responses against oxidative and electrophilic stress, thereby contributing to its antioxidant, anti-inflammatory and neuroprotective effects.^26,27^ In addition, DMF inhibits the nuclear factor kappa B (NF-κB) signalling pathway,^28^ leading to the suppression of cell proliferation, promotion of apoptosis and reduced production of proinflammatory cytokines.^29-31^ Given these immunomodulatory and cytoprotective properties, DMF may have therapeutic applications beyond MS, with the potential to ameliorate the neuroinflammatory pathology of HAM/TSP through distinct mechanisms of action.

Based on these previous findings, we hypothesized that DMF may exert inhibitory effects on cell proliferation and inflammatory cytokine production in lymphocytes derived from people living with HAM/TSP. To test this hypothesis, we conducted an *ex vivo* experiment using peripheral blood mononuclear cells (PBMCs) from people living with HAM/TSP. We evaluated the effects of DMF on cell viability, lymphocyte proliferation, inflammatory cytokine production and HTLV-1 PVL to assess its potential as a novel therapeutic agent for HAM/TSP.

## Materials and methods

### Study participants

In this study, we used PBMC samples from 16 individuals living with HAM/TSP [four men and 12 women; median age, 69.5 years; interquartile range (IQR), 63.0–73.0 years] and five healthy controls (HCs; three men and two women; median age, 43.0 years; IQR, 41.0–52.0 years), cryopreserved at our institution. People living with HAM/TSP were diagnosed according to World Health Organization criteria.^32^ Characteristics of people living with HAM/TSP, including sex, age and disease status, are listed in Table 1. The Osame Motor Disability Score (OMDS) is a clinical scale for assessing motor impairment in people living with HAM/TSP.^15^ At the time of PBMC collection, people living with HAM/TSP were not receiving treatment with glucocorticoids or immunosuppressive agents. PBMCs were isolated from peripheral blood using the Ficoll–Hypaque density gradient centrifugation method, according to the manufacturer’s instructions. The isolated PBMCs were cryopreserved in a freezing medium (Cell Banker 1, Nihon Pharmaceutical Co., Ltd.) and stored in liquid nitrogen until use. The study was approved by the Institutional Review Board of Kagoshima University (protocol no. G491). All participants provided written informed consent before participation, in accordance with the Declaration of Helsinki.

**Table 1:** Clinical and biological characteristics of HAM patients

Subject	Sex	Age (years)	Disease duration (years)	OMDS at sample collection	PBMC PVL (%)
HAM-1	Male	80	6	5	12.9
HAM-2	Female	70	2	2	5.8
HAM-3	Female	69	5	4	5.6
HAM-4	Female	73	4	4	9.1
HAM-5	Female	68	6	4	3.1
HAM-6	Female	83	4	6	4.2
HAM-7	Male	64	5	4	9.1
HAM-8	Female	72	29	11	10.7
HAM-9	Female	67	18	3	0.1
HAM-10	Female	61	5	5	9.2
HAM-11	Female	65	48	11	6.9
HAM-12	Female	62	8	4	8.6
HAM-13	Male	71	5	5	27.7
HAM-14	Male	77	4	5	10.9
HAM-15	Female	51	26	11	11.3
HAM-16	Female	49	10	5	5.2

HAM = HTLV-1-associated myelopathy; OMDS = Osame Motor Disability Score; PBMC = peripheral blood mononuclear cells; PVL = proviral load.

### Cell viability

The PBMCs from five HCs and six individuals living with HAM/TSP were suspended in Roswell Park Memorial Institute (RPMI) 1640 medium containing 10% fetal bovine serum (FBS), 100 U/ml penicillin and 100 μg/ml streptomycin. The cells were then seeded at a concentration of 2.0 × 10^5^ cells/well in a 96-well round-bottom microplate, with DMF concentrations of 0, 5, 10, 25, 50 and 100 µM. PBMCs were cultured in an incubator at 37.0°C with 5% CO_2_ and 95% air. After incubation, the cells were mixed with Trypan Blue solution, and cell viability was assessed on Days 0, 1, 3 and 6 using an automated cell counter (TC20, Bio-Rad Laboratories).

### XTT cell proliferation assay

PBMCs from 10 individuals living with HAM/TSP were suspended in RPMI 1640 medium supplemented with 10% FBS, 100 U/ml penicillin and 100 μg/ml streptomycin. The cells were seeded at a density of 2.0 × 10^5^ cells/well into a 96-well flat-bottom microplate and treated with DMF concentrations of 0, 10, 25, 50 and 100 µM. PBMCs were cultured in an incubator at 37.0°C with 5% CO_2_ and 95% air for 72 h. After incubation, 2,3-bis-(2-methoxy-4-nitro-5-sulfophenyl)-2H-tetrazolium-5-carboxanilide (XTT) labelling reagent was added, and the cells were further incubated under the same conditions for 5 h. Absorbance was measured using an ELISA reader. The absorbance of the produced formazan was recorded at 492 nm, with a reference wavelength set at 690 nm. Specific absorbance was calculated using the following formula: [A492 (test)–A492 (blank)]–[A690 (test)–A690 (blank)]. All experiments were performed in triplicate and mean values reported.

### Cell trace violet cell proliferation assay

PBMCs from 10 individuals living with HAM/TSP and CD3+ CD4+ T cells isolated from PBMCs of eight individuals living with HAM/TSP were labelled with cell trace violet (CTV) (CellTrace Violet Cell Proliferation Kit; Invitrogen), which was dissolved in dimethyl sulfoxide (DMSO) and diluted with PBS. The cell suspension was prepared at a concentration of 1.0 × 10^6^ cells/ml, according to the manufacturer’s instructions. The labelled PBMCs and CD3+ CD4+ T cells were seeded at a density of 2.0 × 10^5^ cells/well into a 96-well round-bottom microplate along with DMF concentrations of 0, 5, 10, 25, 50 and 100 µM. The cells were cultured for 6 days. At Day 0 (before culture) and Day 6, PBMCs were stained with antibodies against CD3, CD4, CD8, CD19, cell adhesion molecule 1 (CADM1) and 7-amino-actinomycin D (7-AAD) (CD3, Beckman Coulter; CD4, BD Biosciences; CD8, CD19 and 7-AAD, BioLegend; CADM1, MBL). CD3+ CD4+ T cells were stained with antibodies against CD3, CD4, CADM1 and 7-AAD. The stained cells were analysed using flow cytometry (FCM). FCM data were acquired using a CytoFLEX flow cytometer (Beckman Coulter) and analysed with FlowJo v.10.10.0 software (FlowJo LLC).

### Flow sorting of CD3+ CD4+ populations

Flow cytometry sorting of CD3+ CD4+ cells from 2 × 10^7^ PBMCs was performed on an SH800 cell sorter (Sony Biotechnology). The antibodies used in the experiments were CD3 and CD4 (CD3, Beckman Coulter; CD4, BD Biosciences). The purity of CD3+ CD4+ T cells was 85.8%–98.8%.

### Measurement of inflammatory cytokines

The concentrations of IFN-γ, interleukin (IL)-2, IL-4, IL-6, IL-10 and tumor necrosis factor (TNF)-α in the supernatants of cultures were measured using a human cytokine magnetic bead panel (Millipore), following the manufacturer’s instructions. PBMCs from 10 individuals living with HAM/TSP were cultured at 2.0 × 10^5^ cells/well into a 96-well plate and treated with DMF concentrations of 0, 5, 10, 25, 50 and 100 µM. The supernatants were collected after 6 days of culture. All samples were analysed on a Multiplex MAGPIX system (Millipore) using the Luminex xPONENT 4.3 software (Millipore; https://int.diasorin.com/en/luminex) for data acquisition. All experiments were conducted in duplicate, and mean values reported.

### Measurement of HTLV-1 proviral loads

HTLV-1 PVL was measured using real-time PCR as previously reported.^33^ PBMCs from 10 individuals living with HAM/TSP were cultured at 2.0 × 10^5^ cells/well into a 96-well plate and treated with DMF concentrations of 0, 5, 10, 25, 50 and 100 µM. The cells were collected after 6 days of culture. DNA was extracted from PBMCs using a DNeasy Blood and Tissue Kit (Qiagen) in accordance with the manufacturer’s instructions. All samples were tested in triplicate, and the average value reported.

### Statistics

The Friedman test, followed by Dunn’s test, was used to compare cell viability, absorbance in the XTT cell proliferation assay, the extent of cell proliferation in the CTV assay, the levels of inflammatory cytokines and the HTLV-1 PVL under different concentrations of DMF treatment. All statistical analyses were performed using Prism version 10.4.2 (GraphPad Software; https://www.graphpad.com). *P*-values of <0.05 were considered statistically significant. Values are presented as medians with interquartile ranges.

## Results

### DMF suppresses the spontaneous lymphoproliferation of PBMCs derived from people living with HAM/TSP

**Figure 1:**
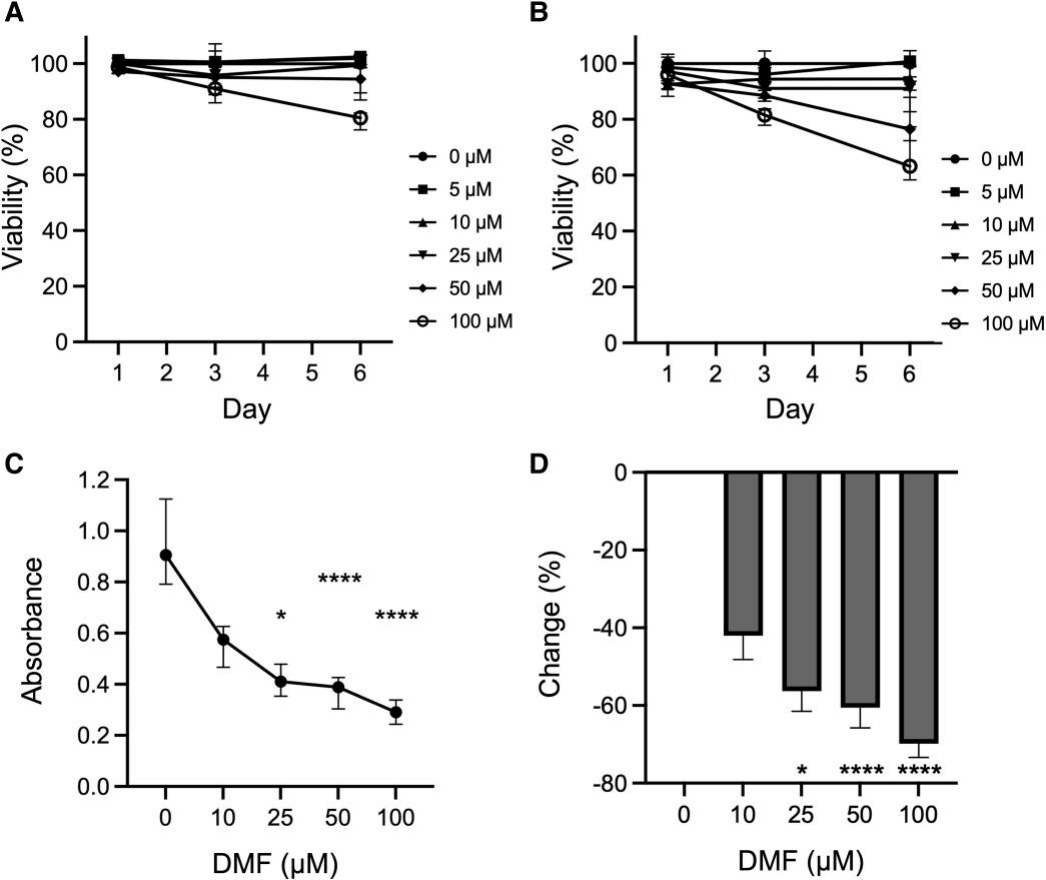
**Inhibition of spontaneous proliferation of PBMCs from people living with HAM/TSP with DMF.** Cell viability of peripheral blood mononuclear cells (PBMCs) from five healthy controls (**A**) and six people living with human T-cell lymphotropic virus type 1 (HTLV-1)-associated myelopathy/tropical spastic paraparesis (HAM/TSP) (**B**) cultured with dimethyl fumarate (DMF). (**C**) DMF exhibits a concentration-dependent inhibitory effect on spontaneous lymphoproliferation in cultured PBMCs from 10 people living with HAM/TSP following a 72-h incubation. Each bar represents the median with interquartile range. (**D**) Per cent change in spontaneous lymphoproliferation in cultured PBMCs from 10 people living with HAM/TSP with DMF compared with those untreated, following a 72-h incubation. Each bar represents the median with interquartile range. *P < 0.05 and ****P < 0.0001.

We first assessed the cytotoxicity of DMF by comparing the cell viability of PBMCs derived from HCs between DMF-treated and untreated groups. After 6 days of culture, DMF at concentrations of 0, 5, 10, 25 and 50 µM had no significant effect on cell viability (Fig. 1A). Treatment with 100 µM DMF showed a mild decrease in cell viability, by 19.5% (range: 16.2% to 25.6%) on Day 6. However, this decrease was not considered sufficient to indicate cytotoxicity, and thus DMF at 100 µM was also regarded as non-toxic. When PBMCs from people living with HAM/TSP were examined under the same conditions, cell viability after 6 days of culture was comparable to that of HCs at 5–25 µM DMF. In contrast, moderate reductions were observed at higher concentrations—by 23.5% (range: 12.2%–27.7%) at 50 µM and 36.8% (range: 22.7%–41.7%) at 100 µM DMF (Fig. 1B).

Since DMF does not significantly affect the cell viability of PBMCs from HCs, we next examined whether DMF at the same concentrations could suppress the spontaneous proliferation of PBMCs derived from people living with HAM/TSP. PBMCs from people living with HAM/TSP exhibit spontaneous *ex vivo* proliferation, reflecting the HTLV-1-driven clonal expansion and immune activation underlying disease pathogenesis.^34,35^ The XTT cell proliferation assay was used to evaluate the inhibitory effects of DMF on spontaneous proliferation. When comparing the absorbance of PBMCs from people living with HAM/TSP cultured with DMF to that of untreated cells, a significant concentration-dependent decrease in absorbance was observed in those treated with DMF (25, 50 and 100 µM) after 72 h of culture (Fig. 1C). Compared with spontaneous lymphoproliferation without DMF, the median percentage reduction among all people living with HAM/TSP was 42.1%, 56.3%, 60.6% and 69.9% at DMF concentrations of 10, 25, 50 and 100 µM, respectively (Fig. 1D). These findings demonstrate that DMF significantly reduces *ex vivo* spontaneous lymphoproliferation in PBMCs from people living with HAM/TSP.

### DMF inhibits spontaneous proliferation of CD8+, CD4+ and HTLV-1-infected cells from people living with HAM/TSP

**Figure 2:**
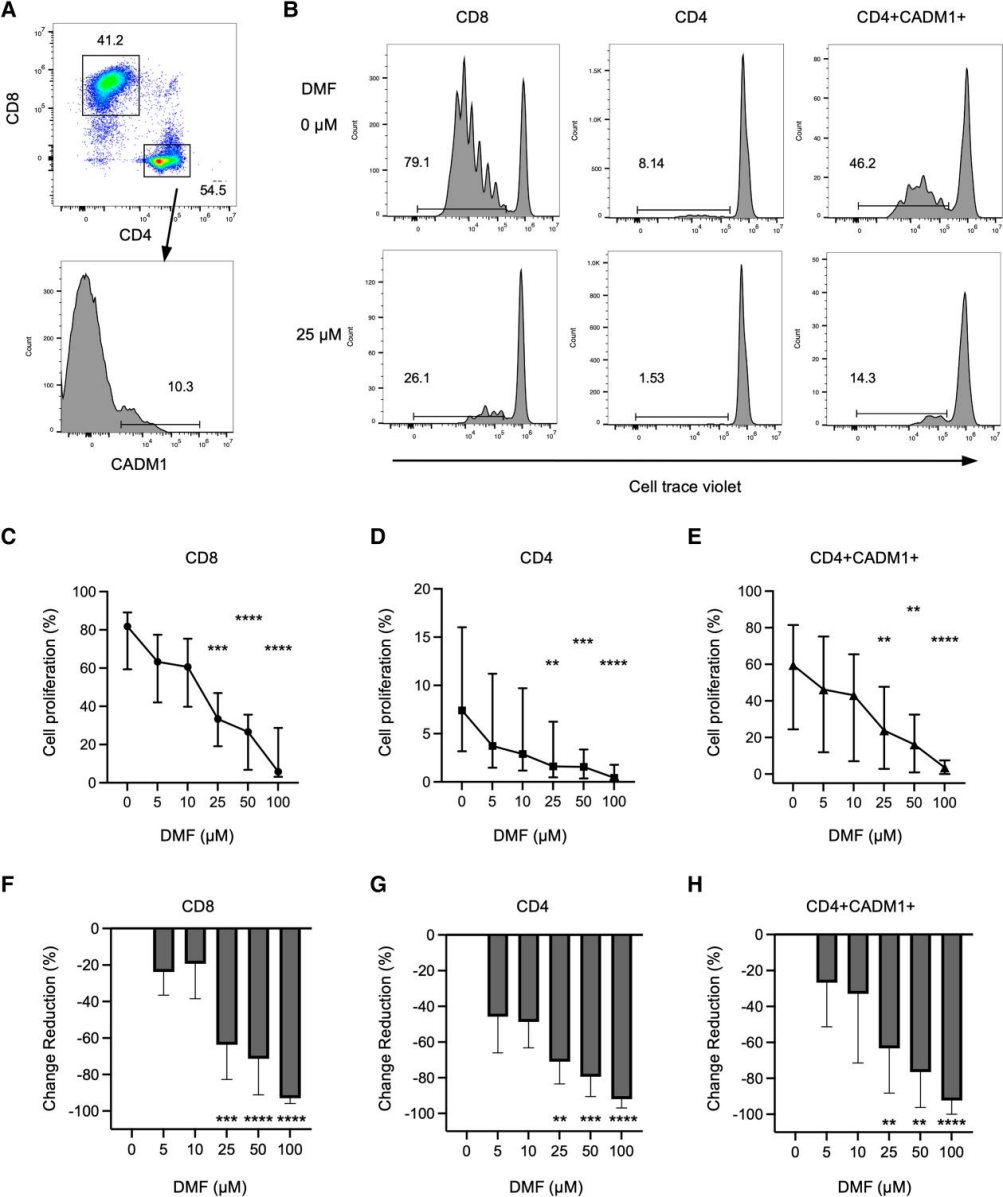
**Inhibition of spontaneous proliferation of CD8+, CD4+ and HTLV-1-infected cells from people living with HAM/TSP with DMF.** (**A**) Gating strategy of CD4+, CD8+ and cell adhesion molecule 1 (CADM1)+ CD4+ T cells from peripheral blood mononuclear cells (PBMCs) of people living with human T-cell lymphotropic virus type 1 (HTLV-1)-associated myelopathy/tropical spastic paraparesis (HAM/TSP). (**B**) Representative dot plots of cell trace violet staining in CD8+, CD4+ and CADM1+ CD4+ T cells from an individual with HAM/TSP (HAM-7) after a 6-day culture with the defined concentration of dimethyl fumarate (DMF). Inhibitory effect of DMF on the proliferation of CD8+ (**C**), CD4+ (**D**) and CADM1+ CD4+ T cells (**E**) in cultured PBMCs from 10 people living with HAM/TSP at Day 6. Each bar represents the median with interquartile range. Percentage change in the proliferation of CD8+ (**F**), CD4+ (**G**) and CADM1+ CD4+ T cells (**H**) in cultured PBMCs from 10 people living with HAM/TSP treated with DMF compared with untreated controls at Day 6. Each bar represents the median with interquartile range. **P < 0.01, ***P < 0.001 and ****P < 0.0001.

Since DMF was able to suppress spontaneous lymphoproliferation in PBMCs from people living with HAM/TSP, we next sought to determine which lymphocyte subset was responsible for this effect. Representative dot plots showing the spontaneous proliferation of CD8+ T cells, CD4+ T cells and CADM1+ CD4+ T cells with or without DMF using CTV are presented in Fig. 2A and B, and Supplementary Fig. 1. The CADM1 antibody is widely used as a surface marker to identify HTLV-1-infected cells.^8,36^ Both CD8+ and CD4+ T cells exhibited spontaneous proliferation, with CD8+ T cells showing a greater extent of proliferation (Fig. 2B), consistent with previous reports.^35,37^ Among CD4+ T cells, the CD4+ CADM1+ subset demonstrated a higher proliferative capacity (Fig. 2B). In cells from a representative individual with HAM/TSP, the addition of 25 µM DMF resulted in a reduction of spontaneous proliferation in CD8+, CD4+ and CADM1+ CD4+ T cells (Fig. 2B). Analysis of CD8+ and CD4+ T-cell proliferation derived from 10 people living with HAM/TSP revealed a significant, dose-dependent reduction following the addition of DMF at concentrations of ≥25 µM (Fig. 2C and D). Notably, DMF at concentrations ≥25 μM was shown to significantly suppress the proliferation of HTLV-1-infected cells within the CD4+ T-cell subset in a dose-dependent manner (Fig. 2E). The median percentage inhibition of highly proliferative CD8+ T cells among all people living with HAM/TSP was 19.3%, 63.7%, 71.4% and 93.0% at DMF concentrations of 10, 25, 50 and 100 µM, respectively (Fig. 2F). In the less proliferative CD4+ T cells, the median percentage suppression was also substantial, reaching 48.8%, 71.0%, 79.5% and 92.0% at DMF concentrations of 10, 25, 50 and 100 µM, respectively (Fig. 2G). Furthermore, DMF exhibited a dose-dependent inhibitory effect on the proliferation of HTLV-1-infected cells, with reductions of 33.0%, 63.3%, 76.5% and 92.4% observed at concentrations of 10, 25, 50 and 100 µM, respectively (Fig. 2H). These results demonstrate that DMF inhibits the spontaneous proliferation of CD8+, CD4+ and HTLV-1-infected cells in *ex vivo* PBMC cultures from people living with HAM/TSP.

### DMF directly suppresses the spontaneous proliferation of CD4+ T cells and HTLV-1-infected cells from people living with HAM/TSP

**Figure 3:**
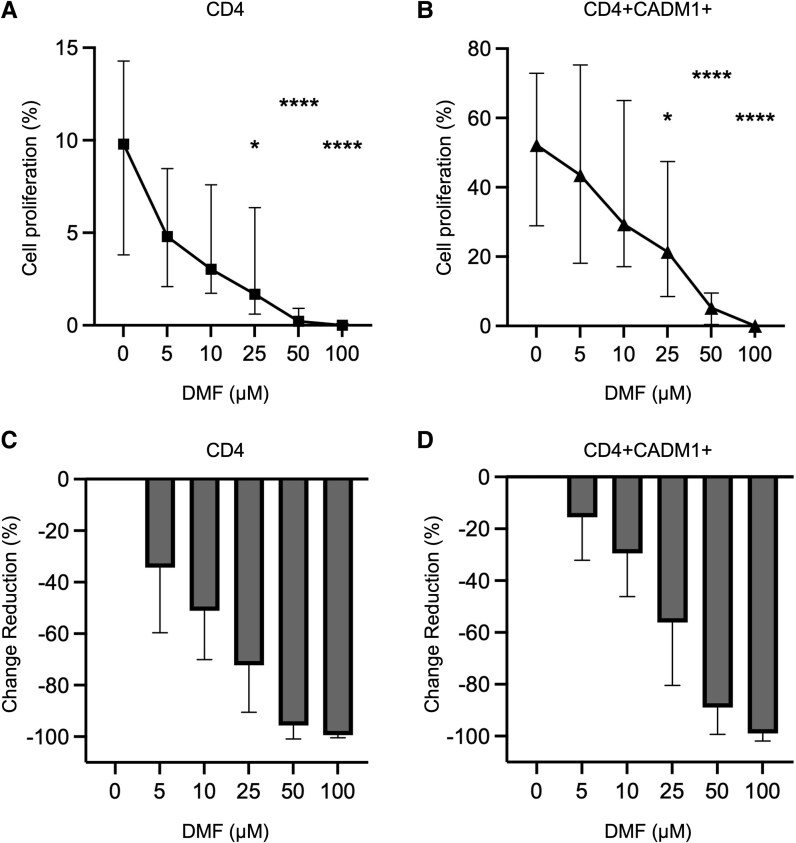
**Inhibitory effect of DMF on CD4+ and HTLV-1-infected cells isolated from people living with HAM/TSP.** Purified CD4+ (**A**) and cell adhesion molecule 1 (CADM1)+ CD4+ (**B**) T cells isolated from peripheral blood mononuclear cells (PBMCs) of eight people living with human T-cell lymphotropic virus type 1 (HTLV-1)-associated myelopathy/tropical spastic paraparesis (HAM/TSP) were cultured for 6 days in the presence or absence of dimethyl fumarate (DMF) to evaluate its inhibitory effect. The percentage change in proliferation of CD4+ (**C**) and CADM1+ CD4+ T cells (**D**) relative to untreated controls after 6 days of culture is shown. Each bar represents the median with interquartile range. *P < 0.05 and ****P < 0.0001.

During spontaneous proliferation of PBMCs from people living with HAM/TSP, CD4+ T cells are known to induce Tax expression and become targets of cytotoxic responses mediated by HTLV-1-specific CTLs. Therefore, it was unclear whether the inhibitory effect of DMF on CD4+ T-cell proliferation resulted from CD8+ T-cell-mediated cytotoxicity or a direct effect of DMF. To address this question, CD3+ CD4+ T cells were isolated from PBMCs of eight individuals living with HAM/TSP and cultured with or without DMF. Consistent with the findings from PBMC cultures described above, spontaneous lymphoproliferation of CD4+ T cells was observed in the absence of DMF, with a higher proliferative rate among CADM1+ CD4+ T cells (Fig. 3A and B). Upon DMF treatment, a significant, concentration-dependent reduction in proliferation was observed in both CD4+ and CADM1+ CD4+ T cells. The median reduction in CD4+ T-cell proliferation was 55.0%, 77.0%, 97.4% and 100% at DMF concentrations of 10, 25, 50 and 100 µM, respectively (Fig. 3C). Similarly, proliferation of CADM1+ CD4+ T cells was reduced by 31.2%, 59.0%, 93.0% and 100% at 10, 25, 50 and 100 µM, respectively (Fig. 3D). These findings demonstrate that DMF directly suppresses *ex vivo* spontaneous proliferation of CD4+ T cells and HTLV-1-infected T cells from people living with HAM/TSP, independent of CD8+ T-cell-mediated cytotoxicity.

### DMF inhibits proinflammatory cytokine production in PBMCs from people living with HAM/TSP

**Figure 4:**
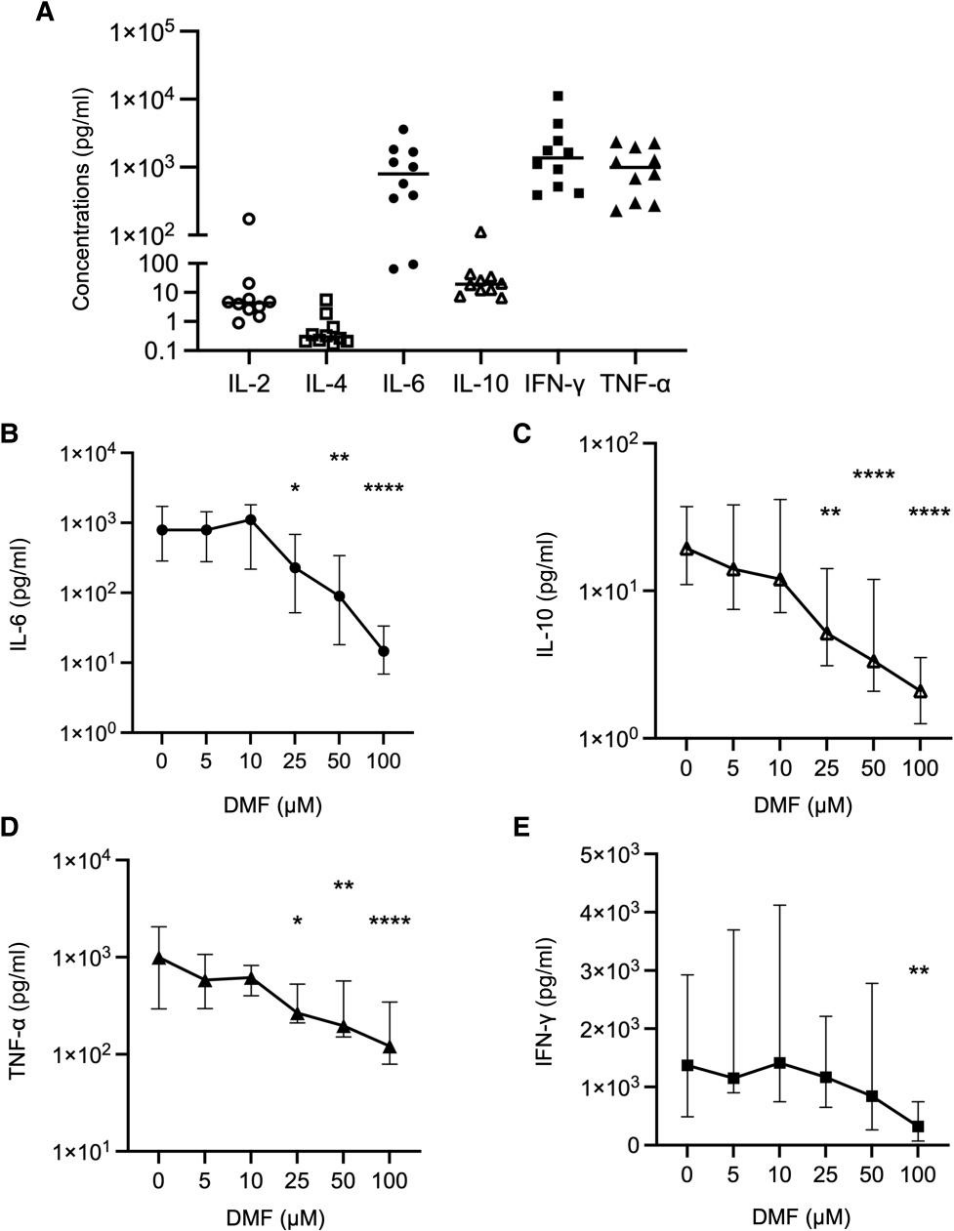
**DMF inhibits cytokine production in PBMCs from people living with HAM/TSP.** (**A**) Direct comparison of the concentrations of cytokines, including interleukin (IL)-2, IL-4, IL-6, IL-10, interferon-gamma (IFN-γ) and tumor necrosis factor-alpha (TNF-α), in the supernatants of untreated peripheral blood mononuclear cells (PBMCs) from people living with human T-cell lymphotropic virus type 1 (HTLV-1)-associated myelopathy/tropical spastic paraparesis (HAM/TSP) cultured for 6 days. Each bar represents the median. (**B–E**) The effects of dimethyl fumarate (DMF) on the concentrations of IL-6 (**B**), IL-10 (**C**), TNF-α **(D)** and IFN-γ (**E**). Each bar represents the median with interquartile range. *P < 0.05, **P < 0.01 and ****P < 0.0001.

Because DMF was found to inhibit the spontaneous proliferation of lymphocytes derived from people living with HAM/TSP, we subsequently examined its effects on the production of inflammatory cytokines. Cytokines released from HTLV-1-infected cells and immune cells, including CNS-infiltrating CTLs, are thought to play a central role in the pathogenesis of HAM/TSP by inducing bystander damage to surrounding neural tissue.^7^ After 6 days of unstimulated culture, PBMCs from people living with HAM/TSP released various proinflammatory cytokines, with notably high levels of IL-6, IFN-γ and TNF-α alongside mild increases in IL-2, IL-4 and IL-10 (Fig. 4A). The addition of DMF (≥25 μM) significantly reduced the concentrations of IL-6, IL-10 and TNF-α (Fig. 4B–D). Furthermore, a significant reduction in IFN-γ levels was observed in cultures with DMF at 100 μM (Fig. 4E). The levels of IL-2 and IL-4, which were not elevated after the culture, remained unaffected by DMF treatment (data not shown). These findings indicate that DMF exerts an inhibitory effect on inflammatory cytokines involved in spontaneous lymphocyte proliferation from people living with HAM/TSP.

### Inhibitory effect of DMF on HTLV-1 PVL in people living with HAM/TSP exhibiting high viral proliferative activity

**Figure 5:**
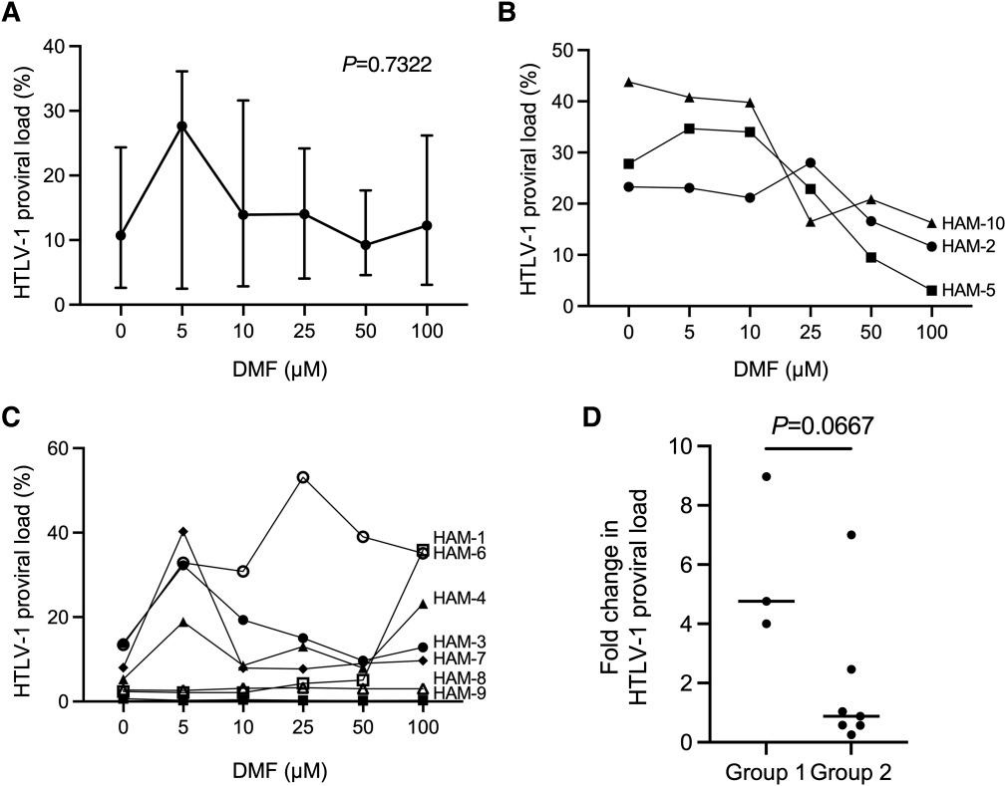
**HTLV-1 PVL in cultured PBMCs from people living with HAM/TSP by DMF.** (**A**) The effect on human T-cell lymphotropic virus type 1 (HTLV-1) proviral load (PVL) in cultured peripheral blood mononuclear cells (PBMCs) from 10 people living with HTLV-1-associated myelopathy/tropical spastic paraparesis (HAM/TSP) with defined concentrations of dimethyl fumarate (DMF). Each bar represents the median with interquartile range. People living with HAM/TSP are classified into two groups based on the change of HTLV-1 PVL in PBMCs treated with DMF. (**B**) Group 1: Individuals showing a decrease in HTLV-1 PVL. (**C**) Group 2: Individuals showing stable or increased HTLV-1 PVL. (**D**) Comparison of the fold change in HTLV-1 PVL after 6 days of culture between Group 1 and Group 2. The fold change was calculated as the ratio of HTLV-1 PVL after culture to that before culture.

Given the inhibitory effect of DMF on the proliferation of HTLV-1-infected cells, we subsequently investigated its potential to reduce the HTLV-1 PVL in *ex vivo* PBMC cultures derived from people living with HAM/TSP. The PVL in PBMCs of people living with HAM/TSP varied from 0.1% to 12.9% (Table 1). The PBMCs derived from 10 people living with HAM/TSP were cultured with or without DMF for 6 days, and the PVL was subsequently quantified. A comparison of PVL revealed no statistically significant differences between DMF-treated and untreated groups at the defined concentrations (Fig. 5A). However, a reduction in PVL following DMF treatment was observed in a subset of *ex vivo* PBMCs cultures derived from individuals living with HAM/TSP. Based on the DMF concentration-dependent changes in HTLV-1 PVL, we classified people living with HAM/TSP into two groups: individuals with decreased PVL into Group 1 (Fig. 5B), and those with stable or increased PVL into Group 2 (Fig. 5C). In Group 1, individuals with a 20% or greater increase in PVL in the absence of DMF exhibited a notable reduction in PVL upon addition of DMF at concentrations above 25 µM (Fig. 5B). In contrast, in individuals of Group 2 with PVL increases of less than 20%, the addition of DMF induced no suppressive effects on the PVL (Fig. 5C). When comparing the two groups, the fold change in PVL after 6 days of culture tended to be higher in Group 1 than in Group 2 (Fig. 5D). Although the sample size was limited and no statistically significant differences were observed in cytokine levels or lymphocyte proliferation between groups, IFN-γ and TNF-α levels tended to be higher in Group 1 under DMF-free conditions (data not shown). These results suggest that DMF may exert an inhibitory effect on PVL in *ex vivo* PBMCs derived from people living with HAM/TSP exhibiting strong proliferative viral activity.

## Discussion

Despite remarkable advances in the treatment of various neurological diseases in recent years, HAM/TSP—first described in the 1980s—still lacks any effective therapy. Identifying a truly effective treatment for this debilitating and intractable condition represents one of the most pressing and unresolved challenges in neurotherapeutics. In this study, we demonstrate that DMF significantly inhibited the spontaneous lymphocyte proliferation of PBMCs derived from people living with HAM/TSP. The suppressive effect of DMF was observed in CD8+ T cells, CD4+ T cells and HTLV-1-infected cells within PBMC cultures, with the most pronounced inhibition seen in CD8+ T cells. In addition, DMF directly suppressed spontaneous proliferation of isolated CD4+ and HTLV-1-infected cells, indicating a direct antiproliferative effect independent of CD8+ T-cell-mediated cytotoxicity. Furthermore, DMF reduced the production of inflammatory cytokines released from these proliferating cells in a concentration-dependent manner.

DMF exerts therapeutic effects in immune-mediated neurological disorders, such as MS, through multiple pathways. DMF has been shown to induce T-cell apoptosis, particularly in CD8+ T cells, and to reduce peripheral lymphocyte counts, reflecting its potent immunomodulatory properties.^38,39^ Intracellularly, DMF depletes glutathione (GSH) through the formation of S-(1,2-dimethoxycarbonylethyl)glutathione (GS-DMS), activating antioxidant pathways including heme oxygenase-1 (HO-1) and suppressing inflammatory cytokine production.^40-42^ Furthermore, DMF modifies Kelch-like enoyl-coenzyme A (ECH)-associated protein 1 (Keap1), leading to the stabilization and nuclear translocation of Nrf2, which promotes the transcription of antioxidant and cytoprotective genes via antioxidant response element sequences.^26,27,43^ These combined actions contribute to the anti-inflammatory and neuroprotective effects of DMF and support its potential utility in modulating immune responses and protecting the CNS.^44,45^

The pathogenesis of HAM/TSP is primarily driven by the proliferation of HTLV-1-infected CD4+ T cells and an exaggerated immune response, predominantly mediated by HTLV-1-specific CTLs. PBMCs from people living with HAM/TSP proliferate spontaneously in the absence of external stimulation. This phenomenon is driven by the clonal expansion of HTLV-1-infected cells and the associated excessive immune response, reflecting the underlying pathogenesis of HAM/TSP and serving as a useful model for evaluating drug efficacy.^37,46^ In the present study, we used this model to examine the effects of DMF, which significantly suppressed spontaneous lymphocyte proliferation, particularly in CD8+ T cells. DMF also inhibited the proliferation of HTLV-1-infected CD4+ T cells, which express the viral Tax protein. Tax constitutively activates the NF-κB signalling pathway, promoting the survival and expansion of infected cells.^35,47,48^

DMF inhibits the NF-κB pathway, thereby modulating inflammation, immune activation and cell proliferation.^49^ Previous studies have demonstrated that DMF suppresses the growth of ATL and HTLV-1-infected cell lines through this mechanism.^30,50^ Given that proliferating HTLV-1-infected CD4+ T cells infiltrate the CNS and induce activation of virus-specific CTLs—subsequently leading to the release of proinflammatory cytokines and bystander neural injury^9-11^—DMF’s ability to inhibit NF-κB signalling may mitigate both immune-mediated neuroinflammation and neuronal damage in HAM/TSP. These findings support the therapeutic potential of DMF in modulating key pathogenic processes in this intractable neuroinflammatory disease.

In addition to its inhibitory effect on spontaneous proliferation, DMF significantly suppressed the production of inflammatory cytokines in cultured PBMCs from people living with HAM/TSP. After 6 days of unstimulated culture, PBMCs from people living with HAM/TSP exhibited markedly elevated levels of IL-6, IFN-γ and TNF-α, while IL-2, IL-4 and IL-10 were only mildly increased. Among these, IFN-γ and TNF-α, predominantly produced by CD8+ T cells in response to HTLV-1-infected cells, are key mediators of bystander-induced damage to neural tissue.^7^ DMF suppressed the production of these cytokines in a concentration-dependent manner, suggesting its potential for attenuating CNS inflammation by modulating the activity of HTLV-1-specific CTLs. A decrease in IL-10—an anti-inflammatory cytokine mainly secreted by regulatory T cells and a major target of HTLV-1 infection^11^—was also observed. This may reflect DMF’s inhibitory effect on the proliferation of HTLV-1-infected cells. These findings align with known anti-inflammatory actions of DMF, including HO-1 induction, Keap1-Nrf2 pathway activation and NF-κB inhibition. Similar cytokine-suppressive effects of DMF have been reported in MS, where treatment leads to reduced levels of IFN-γ-producing effector T cells and proinflammatory cytokines such as IFN-γ and TNF-α.^24,51^ Of note, IFN-γ, produced by CCR4+ HTLV-1-infected T cells in HAM/TSP, promotes astrocytic CXCL10 expression, perpetuating CNS inflammation through a positive feedback loop.^52^ Thus, the ability of DMF to suppress proinflammatory cytokines may have critical implications for mitigating the immunopathogenesis of HAM/TSP.

DMF has been reported to inhibit viral replication through multiple mechanisms. In HIV-infected T cells, it suppresses viral replication by inhibiting NF-κB nuclear translocation and reducing viral transcription.^53^ In the case of severe acute respiratory syndrome corona virus 2 (SARS-CoV-2), DMF reduces viral replication by activating the Nrf2 pathway, enhancing antioxidant responses and limiting proinflammatory cytokine production in lung epithelial cells.^54^ In the present study, we found that DMF significantly suppressed the proliferation of HTLV-1-infected CD4+ CADM1+ T cells. However, no significant change was observed in the HTLV-1 PVL. These findings suggest that DMF may not directly inhibit HTLV-1 replication; instead, it may modulate pathogenic processes such as chronic inflammation and abnormal T-cell proliferation. It should also be noted that the CADM1 antibody was used as a surface marker to identify HTLV-1-infected cells. Its detection sensitivity is approximately 65%.^36^ Therefore, infected cells that did not express CADM1 may have contributed to the unchanged PVL results. Interestingly, a reduction in PVL was observed in a subset of *ex vivo* PBMC cultures derived from individuals living with HAM/TSP who exhibited high viral proliferative activity, indicating that the impact of DMF on PVL may vary between individuals. Further studies in larger cohorts are warranted to clarify the effect of DMF on HTLV-1 PVL.

In this study, we investigated the immunomodulatory effects of DMF on PBMCs from people living with HAM/TSP using concentrations ranging from 0 to 100 µM. Although the upper range exceeds the maximum plasma concentration of its active metabolite, monomethyl fumarate (approximately 13.6 µM) observed *in vivo*,^55^  *in vitro* conditions do not directly reflect systemic pharmacokinetics. Importantly, the lower range (0–25 µM) corresponds to clinically relevant concentrations, and similar ranges (10–100 µM) have been widely employed in *in vitro* studies of MS, demonstrating consistent immunomodulatory and neuroprotective effects.^27,56^ Therefore, the concentrations used in the present study can be considered acceptable and pharmacologically meaningful within the context of *in vitro* experimentation. Furthermore, our additional viability assays demonstrated that DMF up to 50 µM did not affect the survival of PBMCs from HCs, whereas a modest reduction was observed in PBMCs from people living with HAM/TSP at 50–100 µM. These findings suggest that the antiproliferative effects of DMF at lower concentrations are primarily due to cell-cycle arrest rather than cytotoxicity, while higher concentrations may induce apoptotic pathways in HAM/TSP lymphocytes, which are known to be more vulnerable to oxidative stress.^57,58^

In conclusion, our study demonstrated that DMF significantly suppresses the spontaneous proliferation of PBMCs derived from people living with HAM/TSP. The inhibitory effect was particularly pronounced in CD4+ T cells, including HTLV-1-infected cells, as well as in CD8+ T cells; both play central roles in HAM/TSP pathogenesis. Moreover, DMF markedly reduced inflammatory cytokine production from these proliferating cells. These findings suggest that DMF represents a promising therapeutic candidate for the treatment of HAM/TSP.

## Supplementary Material

awaf447_Supplementary_Data

## Data Availability

The data that support the findings of this study are available from the corresponding author upon reasonable request.
