# Supplementary figures and images for: Dimethyl fumarate as a promising therapeutic candidate for virus-associated myelopathy
Source: Brain. 2026 Feb 5;149(7):2550–60. doi: 10.1093/brain/awaf447 (PMC13337227; doi:10.1093/brain/awaf447)

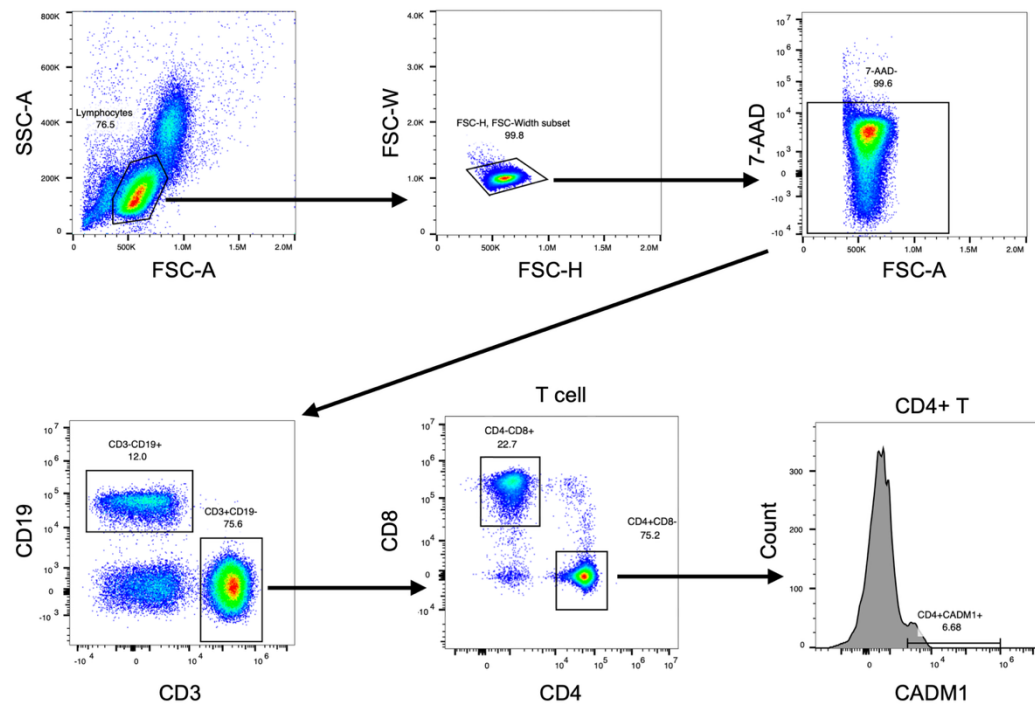

**Figure S1. Gating strategy for CD4+, CD8+, and CD4+CADM1+ cells in cultured PBMCs from patients with HAM/TSP.**

Supplement: awaf447_Supplementary_Data [file awaf447_supplementary_data.pdf]
